# Understanding the Lexicon of Fatigue in Parkinson’s Disease

**DOI:** 10.3233/JPD-202029

**Published:** 2020-07-28

**Authors:** Sneha Mantri, Emily Klawson, Steven Albert, Karina Nabieva, Madeline Lepore, Stephen Kahl, Margaret Daeschler, Eugenia Mamikonyan, Catherine Kopil, Connie Marras, Lana M. Chahine

**Affiliations:** a Department of Neurology, Duke University, Durham, NC, USA; b Department of Behavioral and Community Health Sciences, University of Pittsburgh, Pittsburgh, PA, USA; c The Edmond J Safra Program in Parkinson’s disease, Toronto Western Hospital, University of Toronto, Toronto, Ontario, USA; d Department of Neurology, University of Pittsburgh, Pittsburgh, PA, USA; e Tuck School of Business, Dartmouth College, Hanover, NH, USA; f Michael J. Fox Foundation, New York, NY, USA; g Department of Psychiatry, University of Pennsylvania, Philadelphia, PA, USA

**Keywords:** Fatigue, Parkinson’s disease, qualitative research

## Abstract

**Background:**

Fatigue in Parkinson’s disease (PD) is multifaceted and associated with reduced quality of life. In turn, the language used by people with PD to describe fatigue is variable and poorly understood. We sought to elucidate the lexicon of fatigue using a qualitative grounded theory approach.

**Objective:**

The objective of this study was to understand how patients with PD describe fatigue.

**Methods:**

A pre-study phase of online journaling (Phase 1) provided information regarding topics of importance to patients. Following this, two independent samples of fatigued subjects were studied. Individuals with PD participated in a telephone interview (Phase 2); interview transcripts were analyzed to develop a detailed codebook. To ensure trustworthiness of the findings, an online survey (Phase 3) was administered to individuals with self-reported PD participating in the online study Fox Insight. The survey included the following question: “How do you define fatigue? Please provide your definition in the space below.” The codebook developed from Phase 2 was applied to the Phase 3 responses.

**Results:**

Fifteen individuals participated in Phase 2 and 413 individuals completed Phase 3. Fatigue was subdivided into three domains: cognitive, emotional, and physical. Nearly all individuals experienced more than one domain of fatigue. The most common themes included tiredness, lack of energy, and negative motivation.

**Conclusion:**

Fatigue in PD is multidimensional. Questionnaires that only assess the physical impact of fatigue may not be adequate to capture the broad range of experiences of fatigue among people with PD.

## INTRODUCTION

Fatigue in Parkinson’s disease (PD) has a lifetime prevalence of more than 50% [1], and is a significant contributor to decline in health-related quality of life (HR-QOL) [2]. Despite the breadth of this problem, fatigue in PD remains poorly understood, and there are few effective therapies [3]. The etiology of fatigue in PD is likely multifactorial, including underlying neurodegeneration, other non-motor problems that may manifest as, or be comorbid with, fatigue (sleep problems, depression, apathy), and the effects of PD medications [3]; many patients also experience idiopathic fatigue or fatigue of unknown cause.

Fatigue in PD is heterogeneous not only in its causes but also in its manifestations, encompassing physical, emotional, and cognitive symptoms [4]. Several self-reported questionnaires exist that aim to measure these and other aspects of fatigue, including its functional impact [4–9]; some are PD-specific whereas others have been developed for other patient populations but validated in PD. Several different rating scales have been endorsed by Movement Disorders Society, either as screening measures or for severity rating [7]. While quantitative measures of fatigue are of value, especially in the research setting, their utility in the clinical setting is unclear. In addition, close-ended questionnaires may not capture the entire patient experience and what matters most to patients [10, 11].

A common terminology and taxonomy regarding PD fatigue is important toward progress in understanding and treating it [12]. As this terminology and taxonomy is refined, factoring in the patient’s “voice”, i.e., how patients experience and describe fatigue, is critical toward identifying measures of PD fatigue that are relevant to patients. In this mixed-methods study, we investigated the lexicon of fatigue in PD using a variety of narrative sources by people with PD.

## MATERIALS AND METHODS

### Parkinson’s Disease Education Consortium 2018 Research Program Overview

This work was undertaken as part of the Michael J Fox Foundation’s (MJFF) Parkinson’s Disease Education Consortium (PDEC) 2018 research program. The PDEC objective relevant to the present analysis was to understand how individuals with PD experience fatigue.

**Fig.1:**
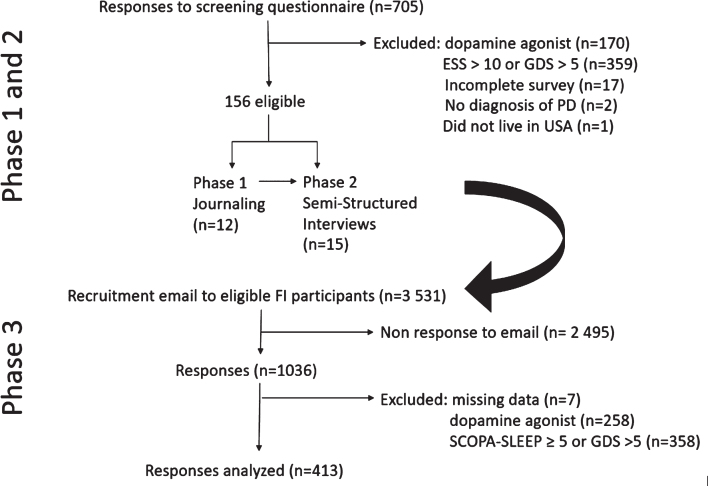
Flow chart of study recruitment phases.

The PDEC 2018 research program undertook a mixed-methods approach that involved three phases (Fig. 1), each of which built on prior phases. Initial phases were aimed at in-depth analysis in a small sample, before expanding to a larger cohort of people with PD. Phase 1 was an online journaling activity, in which an online moderator interacted with individuals with PD via a series of structured activities. Phase 2 involved semi-structured telephone interviews with a different set of participants. Phase 3 involved deployment of a survey to the Fox Insight study cohort. Each of these phases is detailed further below. Participants provided informed consent to each phase separately.

### Study sample and assessments

Phase 1 and Phase 2 Sample Recruitment: Fox Trial Finder (FTF) (https://foxtrialfinder.michaeljfox.org) was used to identify individuals for this phase of the study. As previously described [13], FTF is a database of research volunteers. Individuals enrolled in FTF were sent an email invitation to participate in a study of fatigue in PD. The screening questionnaire included a question on dopamine agonist use, the Parkinson Fatigue Scale (PFS16) [5], Epworth Sleepiness Scale (ESS) [14], and the Geriatric Depression Scale-15 item (GDS) [15]. In an effort to study primary fatigue (i.e., fatigue in individuals who were not depressed or sleepy) participants self-reporting use of a dopamine agonist or with ESS > 10 or GDS > 5 were excluded.

Phase 1 Activities: Online journaling occurred for 1 hour per day over 3 days with a pilot sample of 12 participants (sample size determination was made based on funding and other resources). The online journaling phase consisted of interactive activities including responding to pictures and graphics and completing free-text responses to prompts provided by the research moderator (author CP). Prompts are included in the Appendix. The data collected from phase 1 were informally analyzed by the study team to define dimensions of fatigue important to patients and to inform data collection in other phases.

Phase 2 Activities: Based on the results of the online journaling phase, as well as expert opinion of neurologists with subspecialty expertise in movement disorders (authors CM, LC, SM), a semi-structured interview was drafted and administered over the telephone by research moderator author CP. Questions explored description of fatigue, effect on day-to-day life, barriers to communication with physicians, and perceived triggers and alleviating factors. A full discussion guide is included in the Appendix. A recruitment goal of 15 participants was selected based on available funds; individuals who had participated in Phase 1 were not contacted again for participation in Phase 2. Interviews were audio-recorded, transcribed, and de-identified prior to analysis. The interview transcripts were read in their entirety and analyzed by grounded theory methods to develop a codebook (see below). In response to topics that arose as part of Phase 1, themes were further organized into three domains of physical, cognitive, and emotional subtypes of fatigue.

Phase 3 Recruitment and Activities: The main goal of phase 3 was to study descriptions of fatigue in a large sample of individuals with PD. The sample chosen for phase 3 was a subset of individuals with self-reported PD participating in the Fox Insight study. Fox Insight study methods have been described elsewhere in detail [16]. Briefly, Fox Insight is an online-only longitudinal observational study in which individuals with and without self-reported PD participate in study activities via an online platform. Participants in the PD-arm of the Fox Insight study were invited to participate in this PDEC 2018 sub-study on fatigue in PD if they had completed Fox Insight assessments regarding depression (15-item Geriatric Depression Scale) and Physical Activity Scale for the Elderly (PASE) in the prior 90 days. This latter criterion was determined based on other PDEC 2018 study objectives and to minimize subject burden by utilizing already-existing data that is available from Fox Insight; these scales were not re-administered as part of this study. Eligible participants were invited to take part in this study via email, sent in two waves (March 2019, May 2019).

Assessments in Phase 3, including those administered as part of Fox Insight study (indicated below as “parent FI dataset”) as well as additional questionnaires/surveys collected as part of the PDEC 2018 sub-study, that were considered in this analysis are as follows:

– Demographics (parent FI dataset): age, gender, self-reported year of diagnosis;
– Open-ended question soliciting free-text response on fatigue: “How do you define fatigue? Please provide your definition in the space below”. This was the first question posed to the participant, and allowed the participant an unlimited length of response;
– This open-ended question was followed by the PFS16 and Scales for Outcomes in Parkinson’s disease – Sleep (SCOPA-SLEEP) [17]. SCOPA-SLEEP was administered to identify individuals with daytime sleepiness (see below).

### Codebook development and validation

Transcripts of the semi-structured interviews from Phase 2 were analyzed by researchers trained in qualitative research methods (authors SM, SA, EK). Analysis was blinded to response on the PFS16 or any other part of the FTF screener. NVivo 12 Pro was used to develop a codebook of common themes. Themes were refined by repeated, iterative discussion between researchers [18] until a single standardized codebook was developed. Researchers then categorized themes into three domains based on results of Phase 1: physical, cognitive, and emotional aspects of fatigue; these domains were developed from review of the online journaling components of Phase 1. The Phase 2 codebook was validated by application to the Phase 3 open-ended question on fatigue. As mentioned above, individuals using a dopamine agonist or with significant sleepiness/depression were excluded from phase 2. In order to ensure transferability of the codes between the two cohorts, in turn, in Phase 3 individuals reporting SCOPA-SLEEP≥5, GDS > 5, or use of a dopamine agonist were excluded from this analysis. Responses were classified into one or more themes, which were then tabulated. We compared theme frequency by gender for both Phase 2 and Phase 3.

This study was performed in accordance with the Declaration of Helsinki. This study and the Fox Insight study are approved by the New England Institutional Review Board, and online consent is obtained from each participant at enrollment.

## RESULTS

### Phase 1

Over the 22-day period in which responses on FTF were considered, there were 705 respondents (Fig. 1), of which 170 were on a dopamine agonist; 359 had ESS > 10 or GDS > 5; 17 surveys were incomplete. There were no exclusions based on PFS16 scores. Of the remaining 156 individuals, a consecutive sample was contacted until 12 were recruited to participate in Phase 1. Demographics of this group are shown in Table 1. Nine were female (75%), with a mean age (standard deviation [SD]) of 68 (7.1) years, and a mean disease duration (SD) of 7.4 (5.8) years. As indicated, Phase 1 journals were informally reviewed by members of the study team to ensure that Phase 2 interviews and Phase 3 survey questions incorporated patient-driven concerns.

**Table 1:** Demographic and clinical features for Phases 1 and 2 participants

	ID	Gender	Age (y)	Disease Duration (y)	GDS	ESS	PFS
Phase 1: Online Journaling	1	Female	67	12	2	5	34
	2	Female	59	14	2	6	36
	3	Male	80	5	2	6	55
	4	Female	68	2	1	8	17
	5	Male	69	6	2	6	28
	6	Female	63	7	3	4	50
	7	Male	81	14	1	9	33
	8	Female	68	18	1	4	60
	9	Female	57	0	3	10	56
	10	Male	71	1	1	6	62
	11	Female	66	6	1	NA	40
	12	Male	67	4	1	5	23
Phase 2: Telephone Interviews	13	Male	71	6	2	8	36
	14	Female	72	2	1	5	38
	15	Female	65	2	2	7	56
	16	Male	80	4	2	8	23
	17	Female	77	2	2	2	22
	18	Female	69	5	1	6	61
	19	Female	64	6	2	6	51
	20	Male	78	11	2	5	45
	21	Male	52	3	4	10	65
	22	Male	71	10	3	7	33
	23	Female	66	16	3	9	71
	24	Female	53	4	2	2	17
	25	Female	64	3	2	10	68
	26	Male	68	4	1	9	21
	27	Female	75	5	5	3	49

### Phase 2

Of the 144 eligible respondents on FTF who had not participated in Phase 1, 15 individuals participated in Phase 2. Demographics of this group are shown in Table 1. Nine (60%) were women. Mean age (SD) was 68.3 (8.2) years and mean disease duration (SD) was 5.5 (3.9) years. Mean PFS16 (SD) was 43.7 (18.2). Mean interview duration (SD) was 35.25 (4.5) minutes and covered the topics listed in the Supplementary Material.

### Phase 3

Email invitations for Phase 3 were sent in March and May 2019. In total, 3,531 received an email invitation and 1,036 completed the survey (response rate 29.3%). Seven respondents were excluded for having missing data (age, gender, year of diagnosis). Compared to the remaining 1029, those who did not complete the survey had a longer disease duration (5.34 (5.71) vs 4.60 (5.28), *p* = 0.0002) but did not differ in age, gender, education, or GDS-15 scores. To ensure transferability of codes, 258 responses were excluded for use of a dopamine agonist, and a further 358 were excluded for reporting SCOPA-SLEEP≥5 or GDS > 5, leaving a final sample of 413 surveys (Fig. 1). Demographics for this group are shown in Table 2. Compared to those excluded from analysis, those included were older, had shorter disease duration, were more likely to be women, less likely to be taking levodopa, and had lower PFS16 scores. The mean response length (SD) of Phase 3 free-text responses analyzed in this study was 14.5 (21.0) words.

**Table 2:** Demographics for Responders to Phase 3 invitation. Data is mean (standard deviation) unless otherwise specified. Statistical testing by Pearson’s *χ*^2^ for gender; Student’s *t*-test for all others

	Total sample (*n* = 1029)	Included in analysis (*n* = 413)	Excluded from analysis (*n* = 616)	*p*
Age, y	67.4 (9.3)	68.7 (8.4)	66.5 (9.8)	<0.001
Gender M:F	546:453	211:202	365:251	0.01
Disease duration, y	4.3 (5.3)	3.1 (4.3)	5.1 (5.7)	<0.001
PFS16	48.8 (16.2)	41.3 (15.1)	53.8 (14.9)	<0.001
SCOPA-DS	4.07 (3.3)	2.1 (1.4)	5.4 (3.5)	<0.001
GDS	4.2 (3.7)	2.0 (1.5)	6.8 (4.1)	<0.001

### Theme frequencies and domains

On the basis of Phase 1 journaling responses, fatigue themes were divided into three sub-domains: physical, emotional, and cognitive (Table 3). For example, the physical domain encompassed metaphors of “walking through molasses” or that “every part of [one’s] body weighs a ton.” Cognitive fatigue was noted to be a lack of focus or feeling “not as sharp.” Participants also noted that fatigue impacted their emotional resilience capacity, particularly around negative motivation and stress; one participant reported, “As long as I’m on top of my schedule and things are getting done and I don’t feel stressed, I’m okay. If the waves start going over my surfboard, I’m not surfing along on top of that stress, and the stress is catching up with me, then I do get more fatigued.”

**Table 3:** Themes and frequencies for both study cohorts. Codes for Phase 2 were derived from grounded theory analysis of the entire transcript. Codes for Phase 3 were derived from the free-text response to the following prompt: “How do you define fatigue? Please provide your definition in the space below”

Domain	Theme	Sample phrases	Phase 2 N = 15	Phase 3 N = 413
Physical	Tired	Feeling so tired, I’m having trouble functioning. Feeling uncontrollable ability or uncontrollable tiredness, that no matter how much I sleep it doesn’t go away.	14 (93.3%)	269 (65.1%)
	Lack of Energy	It’s like everything is a lot of effort. Things I’d normally do with quite a bit of ease, I would take a lot of energy, more energy than I really feel like I have to give. The energy is just being erased.	13 (86.7%)	138 (33.4%)
	Exhausted/Depleted	I feel physically spent. Wiped out	10 (67.7%)	56 (13.6%)
	Slowing Down/Dragging	The fatigue that I’ve experienced almost feels like drugged. It feels almost like being in quicksand sometimes. Just walking through molasses	8 (53.3%)	37 (9.0%)
	Heaviness/Weighted	It feels like every part of my body weighs a ton You just cannot move to do something.	7 (46.7%)	10 (2.4%)
	Weakness	A loss of strength It’s my body just needs to be revived somehow.	10 (66.7%)	67 (16.2%)
Cognitive	Overwhelming	Everything requires so much effort that it’s not worth it It’s much deeper and it’s a little more overwhelming at times	9 (60%)	30 (7.3%)
	General Malaise	I’m just going to say, “Wow, I sure don’t feel very good today.” A general sense of not wellbeing	3 (20%)	7 (1.7%)
	Lack of Focus/Blunted	Not able to think clearly It’s harder to find words. My thought processes are not as sharp. Everything about my cognition is dull.	12 (80%)	20 (4.8%)
	Self-Preservation	I don’t trust myself to make the right decision after I get to that spot ... so I wait until I’m more sharp. It makes me self-centered	8 (53.3%)	2 (0.5%)
Emotional	Negative Motivation	I don’t feel like I really want to do anything. Totally unmotivated and like everything is unsurmountable. It can make you feel almost isolated from what’s going on around you because you just can’t participate or do things.	13 (86.7%)	84 (20.3%)
	Positive Motivation	The first thing I try to do is power through As long as I’m on top of my schedule and things are getting done and I don’t feel stressed, I’m okay. If the waves start going over my surfboard, I’m not surfing along on top of that stress, and the stress is catching up with me, then I do get more fatigued. I have to soldier (laugh) through	11 (73.3%)	15 (3.6%)
Other	Other Entity/Mind of its Own	Sometimes it’s waiting for me when we get done with what we’re doing and then I have to go get the rest anyway I think it always wins eventually It just beholds me	10 (66.7%)	17 (4.1%)
	Sudden	I feel like I got bulldozed ... sneaks up on you, so you don’t notice it right away until it hits you	12 (80%)	6 (1.5%)
	Have to Stop	I just can’t do this right now. I’m burnt out. I can’t just soldier through. I really need to just do nothing. I almost have to just actually search out somewhere where I can stop and sit. I can’t even keep moving sometimes. I come up against a brick wall and then I stop	10 (67.7%)	45 (10.9%)

Coded themes and representative quotations from Phase 2 are shown in Table 3. Common themes included tiredness (93.3% of Phase 2, 65.1% of Phase 3), lack of energy (86.7% of Phase 2, 33.4% of Phase 3), and negative motivation (86.7% of Phase 2, 20.3% of Phase 3). Most participants in Phase 2 indicated that the quality of fatigue varied through the day, and 10 of the 15 (66.7%) personified fatigue as an external force over which the subject had little control. By contrast, only 30 of 413 Phase 3 respondents (7.3%) used this metaphor. In comparing theme frequencies by gender (Table 4), women were more likely than men to describe fatigue as overwhelming (*p* = 0.005 for Phase 2, *p* = 0.02 for Phase 3).

**Table 4:** Theme Frequencies by Gender for Phase 2 and 3. *p*-values are by chi-squared analysis

	Phase 2			Phase 3		
	Male N = 6	Female N = 9	*p*	Male N = 211	Female N = 202	*p*
Exhausted or Depleted	4	6	1.0	20	36	0.01
General Malaise	1	2	0.79	3	4	0.66
Have to Stop	3	7	0.26	18	27	0.12
Heaviness or Weighted	1	6	0.06	2	8	0.05
Lack of Energy	5	8	0.76	80	58	0.05
Lack of Focus or Blunted	4	8	0.29	10	10	0.92
Negative Motivation or Apathetic	4	9	0.06	42	42	0.82
Other entity or Mind of its Own	1	6	0.06	9	8	0.88
Overwhelming	1	8	0.005	9	21	0.02
Positive Motivation or Getting Through	6	5	0.06	6	9	0.38
Self-Preservation	2	6	0.21	2	0	0.17
Slowing Down or Dragging	3	5	0.83	15	22	0.18
Sudden	4	8	0.29	4	2	0.44
Tired	5	9	0.21	134	135	0.48
Weakness	4	6	1.0	37	30	0.46

## DISCUSSION

People with PD in this study employed a rich and detailed lexicon around fatigue. In particular, fatigue was determined to be a multidimensional symptom, consisting of emotional components (“overwhelming”), physical sensations (“heaviness”) and cognitive involvement (“fog”, “lack of focus or blunted”). Most individuals experienced more than one form of fatigue during the day. Participants acknowledged a tension between positive motivation (wanting to “power through” their fatigue) and negative motivation. A substantial number of participants described fatigue as an external force, sometimes with anthropomorphic characteristics, which enveloped the subject and against which they struggled.

To our knowledge, this is the first comprehensive qualitative attempt at defining a lexicon of fatigue for people with PD. In doing so, we build on prior smaller studies [21] and anecdotal evidence [22] focusing on the impact of fatigue on people with PD. Currently available fatigue scales emphasize physical and sometimes cognitive domains of fatigue [4–6, 8, 9]; the emotional consequences of fatigue and the anthropomorphic characterization have not been previously noted. Thus, our work uncovers a previously under-recognized aspect of fatigue in PD patient-reported outcomes.

There were marked differences in the frequencies of all themes between cohorts, with the telephone interview group being more likely to report multiple definitions of fatigue than the Phase 3 group; this may be because the detailed and extensive Phase 2 allowed for more nuanced conversation and coding than the relatively shorter responses in the Phase 3. This may also explain why certain themes were much more commonly discussed in Phase 2 than Phase 3 (e.g., positive motivation was discussed by 73.3% of Phase 2 but only 3.6% of Phase 3). Importantly, the difference in modality between a verbal discussion and a typed response may also account for some of the difference in the theme frequency between Phase 2 and Phase 3. Free-text responses acquired on the internet are increasingly a source of qualitative data analysis [19, 20] and allow for rapid collection of data from a diverse and heterogeneous population. Our work demonstrates that direct comparisons between data acquired online and data acquired through traditional means (phone interviews) may prove challenging. A better understanding of how patient descriptions of fatigue may be influenced by the mode of communication is also important for clinical care; for example, descriptions offered electronically over patient portals) may warrant further elaboration during in-person patient visits, especially when fatigue is a big concern for the patient.

Noted strengths of the current study include detailed qualitative analysis, which allowed the development of a rich codebook. Additionally, women with PD, who have traditionally been underrepresented in research, formed a large part of the overall sample; 60% of Phase 2 were female, as were 48.9% of Phase 3. Interestingly, much of the lexicon of fatigue did not differ between men and women in our sample, in contrast with quantitative work suggesting that fatigue is both more common and more severe in women than in men [23]. This may reflect important methodological differences between quantitative and qualitative analyses. We did find that women in both Phase 2 and Phase 3 samples were more likely than men to find fatigue overwhelming. Our findings add to the growing literature on gender differences in non-motor symptoms in PD [23, 24], as well as socially-cued differences between men and women in acknowledging limitations imposed by chronic disease [25].

Our study adds to the development and understanding of a patient-driven lexicon of fatigue in PD. However, some important limitations should be noted. This study recruited individuals participating in the ongoing Fox Insight study. Fox Insight allows for the study of an unprecedented sample size, but it does rely on self-report of PD diagnosis. The Fox Insight cohort are highly educated and motivated to engage in research; this group may not be representative of the PD population at-large. In addition, participants in Phase 2 had to be able to be articulate and audible on the telephone for up to an hour. Individuals with more advanced disease, who often have more hypophonia or cognitive impairment and who may be more vulnerable to fatigue, were therefore largely excluded from the study. Importantly, we attempted to minimize the potentially confounding effects of depression, sleepiness, or use of a dopamine agonist by excluding these individuals from qualitative analysis; however in doing so we may have skewed the study population to a relatively small subset of a complex and heterogeneous disease. Lastly, the online nature of recruitment relies on self-identification of PD, without independent verification of the diagnosis by a movement disorders specialist. Nevertheless, the pattern of responses in the Fox Insight online cohort is consistent with responses derived from traditional in-person cohorts of people with PD [26], suggesting that online self-identification may be a valid way to recruit a global population.

This analysis uncovers important new concepts of fatigue among people with PD. In particular, the cognitive and emotional domains of fatigue are not captured by existing questionnaires such as the PFS16. Other scales, such as the Multidimensional Fatigue Inventory, could be better equipped to capture this aspect of fatigue; assessing the psychometric properties of multiple fatigue scales was outside the scope of the current study. Awareness of the multiple aspects of fatigue may be useful in the development of future questionnaires and surveys that more comprehensively capture the experience and severity of fatigue in this patient population. Additionally, the present work can be used to develop patient-facing materials, as well as provider-facing education. Ongoing analysis from the larger PDEC study will assess whether clinical or demographic features affect measures of fatigue impact and severity, as well as incorporate an analysis of other non-motor symptoms to help contextualize the present work.

## CONFLICT OF INTEREST

Funding source: Michael J. Fox Foundation for Parkinson’s Research.

Conflicts of interest: none.

## Financial disclosures for the past 12 months

SM receives research support from the Michael J Fox Foundation (MJFF), the Parkinson Foundation (PF) and Cerevel Therapeutics, was a paid consultant to MJFF, is a study site investigator for a study sponsored by Neuraly Rho, is a study site sub-investigator for a study sponsored by Biogen, and is contracted with Deep Brain Innovations, LLC. MD was an employee of the sponsor, MJFF, at the time this work was done. CK is an employee of MJFF. CM was a paid consultant for Acorda Therapeutics, on the advisory board of Denali therapeutics, received honoraria for teaching from EMD Serono, a steering committee member for MJFF Grants Canadian Institutes of Health Research, Parkinson’s Foundation (US), National Institutes of Health (US), International Parkinson and Movement Disorders Society, and is contracted with Grey Matter Technologies. LC receives research support from MJFF, has received travel payment from MJFF to MJFF conferences, is a paid consultant to MJFF, receives research support from the UPMC Competitive Medical Research Fund, is study site investigator for a study sponsored by Biogen, is a site sub-investigator for a study sponsored by Voyager, received payment from Elsevier (for book authorship), and receives royalties from Wolters Kluwel (for book authorship). All other authors have no financial disclosures.

## Supplementary Material

Supplementary Material
