# Supplementary material for: Understanding the Lexicon of Fatigue in Parkinson’s Disease
Source: J Parkinsons Dis. 2020 Jul 28;10(3):1185–93. doi: 10.3233/JPD-202029 (PMC7458521; doi:10.3233/JPD-202029)
Supplement: Supplementary Material [file jpd-10-jpd202029-s001.pdf]

Supplementary Material

Understanding the Lexicon of Fatigue in Parkinson's Disease

ONLINE JOURNAL PROMPTS

Video Introduction: Welcome, Overview, Ground Rules, Introductions

Objective: Introductions, ground rules and purpose of the research. Chelle's video will call out basic instructions as well as the instructions about answering each section of the journal.

Intro: Hello, my name is Chelle. I am an independent market researcher – I have been hired as a moderator to guide our conversation and elicit your feedback.

Thank you for being part of this journal! First of all, let me start by thanking you for agreeing to log in two or more times each day for the next three days. We do journals like this so we can understand your thoughts, feelings and experiences at different points of time on different days. This helps us obtain a richer and more nuanced understanding of your experience. Progress in our understanding of Parkinson's disease has been possible because of people who participate in research like this. So, thank you for being part of the research process and sharing your experiences and thoughts today.

No wrong answers: Please know that there's no wrong answers. All we ask is that you answer honestly and thoughtfully.

Background/objective of the groups: We are going to focus on finding out more about your thoughts and feelings around your experience with Parkinson's. Each time you log in (again, twice a day, about 15 to 20 minutes each time you log in, for three days), there will be new questions / exercises to complete. The goal is to get as much information on each topic as possible, so please make sure to be as explicit as possible and answer each question / fill out each exercise as in-depth as you can, please.

Reporting: We are conducting journals with several of you at the same time, and when this is finished we will put together a report based on everything we've learned from all the journals. To assure your confidentiality, no last names are ever included in the report – it's reported as group findings.

Explanation of Journal / Beginning for Each Exercise: I will be logging into the journal each day and will read through your responses. Should I have any questions or need any further clarification, I will leave follow-up questions for you.

If you have any questions, just let me know and I'll answer them the next time I log in.

DAY 1 / Section 1: INTRODUCTION AND BASELINE INFORMATION

Objective: Learn the descriptions / feelings / emotional language they use for fatigue.

Self-Introduction

How old are you now?

At what age were you when you were diagnosed with Parkinson's Disease?

With what gender do you most closely identify: Male or Female?

Question and Answer re: thoughts on fatigue

We're going to be talking a lot about fatigue during the next several days. Before we get going, how do you define fatigue? (Chelle to probe heavily re: tired, sleepy and fatigue.)

I've seen fatigue described as "an abnormal and excessive lack of energy."

What do you think about this definition of fatigue? What terms do you use for what I described here?

So that we're using the same definition for the rest of the journal, when I say fatigue, I mean "an abnormal and excessive lack of energy." Describe how you feel when you are experiencing fatigue.

Describe what the difference is between sleepiness and fatigue, if there is one for you.

What are the symptoms / signs that you are sleepy?

What are the symptoms / signs that you are experiencing fatigue?

Describe what the difference is between fatigue and apathy, or a lack of motivation to do something, if there is one for you.

What are the symptoms / signs that you are lacking motivation?

What are the symptoms / signs that you are experiencing fatigue?

What are all the areas of your life that are impacted by fatigue, if any? Be as descriptive as possible, include as many examples as you can think of, please.

For this next question and throughout the rest of the journal, when I mention cognitive, I'm meaning your thinking, memory, reasoning, attention or concentration. If I were to break down fatigue into two categories, physical and cognitive ….

How would you define physical fatigue? (Be as descriptive as you can including, if you experience physical fatigue, examples of how you feel when you are physically fatigued.)

How would you define cognitive fatigue? (Be as descriptive as you can including, if you experience cognitive fatigue, examples of how you feel when you are cognitively fatigued.)

Please describe the difference, if any, between being physically fatigued and being cognitively fatigued for you.

Assuming that you have both physical and cognitive fatigue, do you ever have one without the other or do they always happen at the same time? Please give examples and be as specific as

possible. (If you don't experience both, just write "don't experience" and tell me which you don't experience.)

Are there any categories of fatigue I missed (in addition to physical and cognitive)? If so, what are they and describe them to me including examples, please?

What about being emotionally fatigued (meaning emotionally drained or exhausted), is that something you experience? If yes, please describe and give me examples. If not, just write in "don't experience."

What do you do when you are experiencing fatigue? Please be as descriptive as possible and explain any different circumstances (meaning if the answer is "it depends" tell me all the different situations and how you respond to each.)

How does that differ from when you aren't experiencing fatigue?

BONUS SECTION – IN THE MOMENT FATIGUE

Objective: Learn the descriptions / feelings / emotional language they use for fatigue while experiencing fatigue at that moment.

If you are taking medication for your Parkinson's Disease, would you describe yourself as being OFF or ON right now? For the purposes of this study, we are going to refer to the time when your medication is doing its best as "ON periods" and when the medication isn't controlling symptoms as well as "OFF periods." (If you aren't taking medication for your Parkinson's, just write in "N/A" please.)

Please sign into the journal and fill out this section when you are ACTUALLY experiencing fatigue.

How do you describe what you are physically feeling?

How do you describe what you are emotionally feeling (meaning emotionally drained or exhausted)?

How do you describe what you are feeling cognitively (meaning thinking, memory, reasoning, attention or concentration)?

DAY 1 / Section 2: COLLAGE

Objective: Projective technique to get more descriptions / feelings / emotional language they use for fatigue.

If you are taking medication for your Parkinson's Disease, would you describe yourself as being OFF or ON right now? For the purposes of this study, we are going to refer to the time when your medication is doing its best as "ON periods" and when the medication isn't controlling symptoms as well as "OFF periods." (If you aren't taking medication for your Parkinson's, just write in "N/A" please.)

Collage: For this exercise, I want you to be creative. I'd like for you to create a collage that best describes fatigue using the pictures provided. I want to know how you feel when you have fatigue, how it impacts your day, how you feel about experiencing it, how often you experience it, everything about fatigue ~ using pictures and words. [USE THE BUILT IN COLLAGE TOOL FOR THIS EXERCISE.]

Give the collage a title: Knowing that the collage is describing fatigue, what would you title the picture to best describe it to me?

Describe why this best describes fatigue: Knowing that I'm not experiencing what you experience each time you experience fatigue, be explicit about describing why the picture

represents you / your fatigue and make sure to answer EACH OF THESE QUESTIONS about the collage in your description, please:

Why does the collage best represent fatigue to you?

Describe each picture and why you used it to describe fatigue.

What emotions is it representing that you are feeling during times when you are fatigued?

How does fatigue impact your day?

How often do you experience fatigue? (Chelle to probe for hours per day and/or days per week.)

What isn't the collage telling me about what you are feeling during times when you are experiencing fatigue?

One word: If you had to pick just one word (or short phrase) that best describes your fatigue what would it be?

Please explain why you chose that word to best describe fatigue for you?

DAY 2 / Section 1: FATIGUE EXPERT LETTER

Objective: Learn the descriptions they use for fatigue by writing a letter to someone who has no idea what Parkinson's is.

If you are taking medication for your Parkinson's Disease, would you describe yourself as being OFF or ON right now? For the purposes of this study, we are going to refer to the time when your medication is doing its best as "ON periods" and when the medication isn't controlling symptoms as well as "OFF periods." (If you aren't taking medication for your Parkinson's, just write in "N/A" please.)

Pretend you have met someone who has never heard of Parkinson's and they don't know about the fatigue you experience. You are going to write a letter to that person and tell them about your

fatigue. Help them understand all about what it is like for you, the differences between when you are experiencing fatigue and when you aren't, what you feel during the times you experience fatigue, how you know when fatigue is happening, etc. Be as descriptive as you can so the person can be an expert about how you experience fatigue by the time they finish your letter.

Start the letter with "Dear ____________" (you fill in the blank). I'm writing this letter to make you an expert on fatigue, often experienced by those who have Parkinson's."

In the body of the letter, make sure to answer EACH OF THESE QUESTIONS in your letter to this person:

How do you feel physically when you are experiencing fatigue?

How do you feel emotionally you are experiencing fatigue?

How do you feel cognitively (for example: your thinking, memory, reasoning, attention or concentration) when you are experiencing fatigue?

What impact does fatigue have on you / your day; what does it do to or for you?

How often are you experiencing fatigue in a typical day/week?

How long does fatigue generally last?

How do you know when fatigue is about to happen?

How do you know the difference between fatigue and other symptoms (such as sleepiness, lack of motivation, etc.), if there is a difference to you?

Close the letter with any additional thoughts that person needs to know so they can be an expert about fatigue from your perspective.

DAY 2 / Section 2: FILL IN THE BLANK

Objective: Understand the severity and how bothersome fatigue is for these patients.

If you are taking medication for your Parkinson's Disease, would you describe yourself as being OFF or ON right now? For the purposes of this study, we are going to refer to the time when your medication is doing its best as "ON periods" and when the medication isn't controlling symptoms as well as "OFF periods." (If you aren't taking medication for your Parkinson's, just write in "N/A" please.)

Fill in the blank: For this next exercise, I want you to fill in the blanks. As we've requested, please remember to be as thorough and descriptive as possible with your answers. The more information you can share, the better it is for us to learn.

When physical fatigue sets in, physically I'm experiencing _______.

When cognitive fatigue sets in (meaning thinking, memory, reasoning, attention or concentration), cognitively I'm experiencing _______.

When emotional fatigue sets in (meaning emotionally drained or exhausted), emotionally I'm experiencing _______.

Fatigue makes me feel like _______.

People who are with me when I'm experiencing fatigue feel _______.

The difference between experiencing fatigue at home vs. experiencing fatigue outside of the home for me is _______.

Fatigue impacts my social life by _______

If I was to rate how bothered I am by fatigue, I'd say it's _______.

Overall, I think fatigue is _______.

On a scale from 1 to 10 where 1 is not at all bothersome and 10 is the most bothersome thing I've experienced in my life, I would say fatigue rates a _______. (If the answer is "it depends,"

feel free to give different ratings for different situations, but make sure to describe the different situations thoroughly!)

Please explain why you gave it that bothersome rating.

Again, on a scale from 1 to 10 where 1 is not at all impactful to my life and 10 is the most impactful thing I've experienced in my life, I would say fatigue rates a _______.

Please explain why you gave it that impactful rating.

My social life is impacted by fatigue like this _______.

My leisure activities are impacted by fatigue like this _______.

My hobbies are impacted by fatigue like this _______.

My work life is impacted by fatigue like this _______.

My relationships are impacted by fatigue like this _______.

My productivity is impacted by fatigue like this _______.

My daily living is impacted by fatigue like this _______.

DAY 3 / Section 1: THINK VS. SAY

Objective: Understand the severity and how bothersome fatigue is for these patients as well as get at communication patterns and barriers between patients / caregivers / physicians.

If you are taking medication for your Parkinson's Disease, would you describe yourself as being OFF or ON right now? For the purposes of this study, we are going to refer to the time when your medication is doing its best as "ON periods" and when the medication isn't controlling symptoms as well as "OFF periods." (If you aren't taking medication for your Parkinson's, just write in "N/A" please.)

There are many aspects of our lives where we think one thing, describe it to our close family one way, our friends another way and then our doctor's even another way. On a completely non-related subject, as an example: let's say a coworker is wearing a red outfit that isn't very flattering. In your mind you might be thinking "wow, that outfit is way too tight" but to your close family / friends you might say "that really isn't the best outfit for her to wear" but to other coworkers you might say "red is really a good color on her."

Thinking about all the ways you think about and talk about fatigue, I'd like for you to answer the questions in this picture:

If there is a difference between what you say to each group: Why do you talk about it differently to your family/ friends/ doctor? As well as to probe if differences between cognitive and physical fatigue for any of their responses.

If I was a doctor and I was trying to learn how to talk with Parkinson's patients about fatigue, what would you tell me to help me best understand?

What questions should I ask?

What wording should I use when I talk about fatigue?

What details should I be aware of that would help me better relate to the patient?

Is there anything that would help with relief?

What would make you more open to discussing your fatigue with me? What could I do?

DAY 3 / Section 2: FATIGUE SYMPTOMS

Objective: Learn the symptoms that happen during periods of fatigue, so we can probe for the language used around them, then rank them by most to least often experienced.

If you are taking medication for your Parkinson’s Disease, would you describe yourself as being OFF or ON right now? For the purposes of this study, we are going to refer to the time when your medication is doing its best as “ON periods” and when the medication isn’t controlling symptoms as well as “OFF periods.” (If you aren’t taking medication for your Parkinson’s, just write in “N/A” please.)

If you take medication for PD, do you notice any difference between the fatigue when you first take your medicine vs. when the medicine is wearing off?

Pick a Picture: For this next exercise, I want you to once again be creative. I’d like for you to pick a picture that best describes how you feel when you are experiencing fatigue.

Give the picture a title: Knowing that the picture represents you when you are experiencing, what would you title the picture to best describe it to me?

Describe why this best represents you when you are experiencing fatigue: Knowing that I’m not experiencing what you experience when you experience fatigue, be explicit about describing why the picture represents when you are experiencing fatigue and make sure to answer EACH OF THESE QUESTIONS about the picture in your description, please:

Why does the picture best represent you when you are experiencing fatigue?

What emotions is it representing that you are feeling during when you are experiencing fatigue?

What isn’t it telling me about what you are feeling when you are experiencing fatigue?

Awards: For this next exercise, we are going to have you issue the following awards:

Least bothersome fatigue you’ve experienced in the past 7 days

Most bothersome fatigue you’ve ever experienced

Most bothersome fatigue in the past 7 days

For each award, please describe to me what made that experience of fatigue fit that award most. Please be as descriptive about the fatigue as possible and answer or describe the following questions in your answer:

Why was that experience of fatigue the one that fit that award best?

How were you feeling at the time? (Include physical, cognitive, and emotional feelings)

Where were you (at home / away from home)?

What was going on at the time / what were you doing?

How did it affect your activity / plans, if at all?

Was there something that brought the fatigue on?

What did you do about it?

Did that resolve the fatigue?

What else do you want to tell me about fatigue that you think I should know about?

SEMI-STRUCTURED INTERVIEW GUIDE, ADMINISTERED OVER TELEPHONE BY CP

I. Welcome, Overview, Ground Rules, Introductions (5 min)

A. Introduction of moderator CP, thanking participant for agreeing to call, no wrong answers. Background/objective of the research project. Anonymity of responses.

B. Any questions before we begin?

II. Medication & Introduction (5 min)

A. Are you currently taking any medication for your Parkinson's Disease?

B. Would you describe yourself as being ON or OFF right now?

C. Demographics: age, disease duration, gender.

III. Fatigue / Sleepy Descriptions (15 min)

A. How do you define fatigue? (CP to probe re: tired, sleepy and fatigue)

B. I've heard mention that there is a difference between fatigue related to Parkinson's Disease and fatigue you might experience that is not related to PD. Do you agree with that, or not? IF SO: what is the difference? Can you give me some examples?

C. Would you say there is a difference between PD related fatigue and the fatigue you had prior to having Parkinson's?

D. I've seen fatigue described as "an abnormal and excessive lack of energy." What do you think about this definition of fatigue? What terms or phases do you use for when you are experiencing "an abnormal and excessive lack of energy"?

F. Describe how you feel when you are experiencing fatigue.

G. What are the symptoms / signs that you are experiencing fatigue vs. being sleepy?

H. If I were to break down fatigue into two categories, physical and cognitive ....

a. How do you feel when you are experiencing physical fatigue?

b. How do you feel when you are experiencing cognitive fatigue?

J. Assuming that you have both physical and cognitive fatigue, do you ever have one without the other or do they always happen at the same time? (CP to probe for examples of physical vs. cognitive.)

K. Are there any categories of fatigue I missed, in addition to physical and cognitive? If so, what are they? How do you feel when you are experiencing this type of fatigue?

L. What about being emotionally fatigued (meaning emotionally drained or exhausted), is that something you experience? IF SO: How do you feel when you are experiencing emotional fatigue?

IV. Fatigue Impact (10 min)

A. What are all the areas of your life that are impacted by fatigue, if any? How does this compare with other PD symptoms?

B. How bothersome is fatigue for you and your everyday life?

C. Thinking of all the symptoms you have with your Parkinson's, where would you rate fatigue in terms of being bothersome? (CP to probe which symptoms are more bothersome and which are less bothersome.)

D. Thinking of all types of fatigue: what do you do when you are experiencing fatigue, meaning how do you solve or help get rid of fatigue? Are there differences in how you react to fatigue for

different situations or is it always the same? (CP to probe for what differences are if they exist and how handle each.)

E. How does fatigue interact, if at all, with your other PD symptoms? (CP, if necessary, does it make other symptoms worse or more difficult to manage, or not really?)

G. Have you noticed any relationship between fatigue and your PD medication, or not? Do you notice fatigue when your PD medication is wearing off or after you’ve just taken your medication? (CP to probe on relationship between fatigue and ON and OFF periods)

V. Fatigue with Physician (3 min)

A. How often do you discuss fatigue with your doctor?

a. IF DISCUSSED: Walk me through a typical discussion with your doctor. When you are talking to the doctor about fatigue, how do you convey the magnitude that fatigue is impacting you? Give me examples of how you discuss the impact, please. Do you initiate the discussion yourself about fatigue with the doctor? If so, tell me about why you bring it up. How do you feel your doctor reacts to discussions about fatigue? (CP: if necessary, ask if the doctor is interested, dismissive, or some other reaction?)

b. IF NOT DISCUSSED: Is there a particular reason you do not discuss fatigue with your doctor? What, if anything, would make you more open to discussing fatigue with your doctor?

B. If I were a doctor and I was trying to learn how to talk with Parkinson’s patients about fatigue, what would you tell me to help me best understand?

C. And still pretending that I’m a doctor talking with Parkinson’s patients, what questions should I ask you about fatigue?

VI. Terminology & Final Questions (7 min)

A. I'd like for you to complete this sentence: Fatigue feels like (......).. If necessary (if answer above is long): If you had to pick just one word or short phrase that best describes your fatigue what would it be? Why did you choose that / what about that best represents fatigue?

B. What else do you want to tell me about fatigue that you think I and doctors who are working with Parkinson's patients should know about?
